# Contextual expectations shape cortical reinstatement of sensory representations

**DOI:** 10.1523/JNEUROSCI.2045-21.2022

**Published:** 2022-06-24

**Authors:** Alex Clarke, Jordan Crivelli-Decker, Charan Ranganath

**Affiliations:** 1 Department of Psychology, University of Cambridge, UK; 2 Center for Neuroscience, University of California, Davis, USA; 3 Department of Psychology, University of California, Davis, USA

## Abstract

When making a turn at a familiar intersection, we know what items and landmarks will come into view. These perceptual expectations, or predictions, come from our knowledge of the context, however it’s unclear how memory and perceptual systems interact to support the prediction and reactivation of sensory details in cortex. To address this, human participants learned the spatial layout of animals positioned in a cross maze. During fMRI, participants of both sexes navigated between animals to reach a target, and in the process saw a predictable sequence of five animal images. Critically, to isolate activity patterns related to item predictions, rather than bottom-up inputs, one quarter of trials ended early, with a blank screen presented instead. Using multivariate pattern similarity analysis, we reveal that activity patterns in early visual cortex, posterior medial regions, and the posterior hippocampus showed greater similarity when seeing the same item compared to different items. Further, item effects in posterior hippocampus were specific to the sequence context. Critically, activity patterns associated with seeing an item in visual cortex and posterior medial cortex, were also related to activity patterns when an item was expected, but omitted, suggesting sequence predictions were reinstated in these regions. Finally, multivariate connectivity showed that patterns in the posterior hippocampus at one position in the sequence were related to patterns in early visual cortex and posterior medial cortex at a later position. Together, our results support the idea that hippocampal representations facilitate sensory processing by modulating visual cortical activity in anticipation of expected items.

## Introduction

Our knowledge of how the world is structured has a powerful influence on our perceptions (Bar, 2004; Oliva and Torralba, 2007; Summerfield and Egner, 2009), allowing predictions of future states we may encounter (Bar, 2009; Eichenbaum and Fortin, 2009). Perceptual expectations, or predictions, take different forms, with one distinction being between predictions dependent on temporal continuity (e.g. a train moving from one location to another as it’s driven), and predictions relating to upcoming items that are not in view. This latter form of perceptual expectation must depend on coordinated responses between perceptual and contextual memory systems, however the neural mechanisms of how memory systems might predict and reactivate perceptual details in cortex is elusive.

Expecting to see a specific visual stimulus generates responses in early visual cortex that resembles activity when perceiving the same stimulus (Kok et al., 2012, 2014), even if the expected item is never shown (Eagleman and Dragoi, 2012). To make accurate predictions requires we’ve encountered similar situations and learned what is likely to occur next. Statistical learning approaches suggest we acquire knowledge about how the world is structured through repeated experiences (Schapiro et al., 2017; Sherman et al., 2020), with this information utilized to guide predictions of future states (Stachenfeld et al., 2017; Turk-Browne, 2019; Barron et al., 2020). In humans, the hippocampus represents predictions of future states (Eichenbaum and Fortin, 2009; Brown et al., 2016), suggesting it might be a primary source of top-down predictions to reactivate expected sensory details. This is supported by recent evidence from Kok and Turk-Browne (2018), who showed that hippocampal responses reflected the expected stimulus following a predictive auditory cue, whilst visual regions reflected the perceived stimulus. Such studies are beginning to reveal how memory and perceptual systems support prediction and perception, highlighting the hippocampal top-down effects on cortex. While learned prior contexts must guide the prediction of future states, direct empirical support for such a contextually dependent hippocampal-cortical interaction remains limited.

Beyond primary sensory regions and the hippocampus, a network of posterior brain regions are implicated in item and context-based reactivations, including the parahippocampal cortex, precuneus/posterior cingulate cortex and angular gyrus (Bar and Aminoff, 2003; Lee and Kuhl, 2016; Livne and Bar, 2016; Jonker et al., 2018; Caplette et al., 2020). This posterior medial (PM) network (Ranganath and Ritchey, 2012; Ritchey and Cooper, 2020) displays connectivity with the posterior hippocampus (Barnett et al., 2019, 2021). In contrast, the anterior hippocampus displays connectivity with an anterior temporal (AT) network including the perirhinal cortex, temporal pole, amygdala and orbitofrontal cortex, linked to item and object information (Ranganath and Ritchey, 2012; Ritchey and Cooper, 2020). The differing connectivity profiles of anterior and posterior hippocampus are linked to differential functional properties (Poppenk et al., 2013), leading us to predict that contextually-driven reactivations will be present in the posterior hippocampus and PM network, with the reactivation of sensory details in primary visual cortex. A further unexplored question is how the hippocampus, PM network and visual cortex interact to support the prediction and reactivation of sensory patterns.

**Figure 1:**
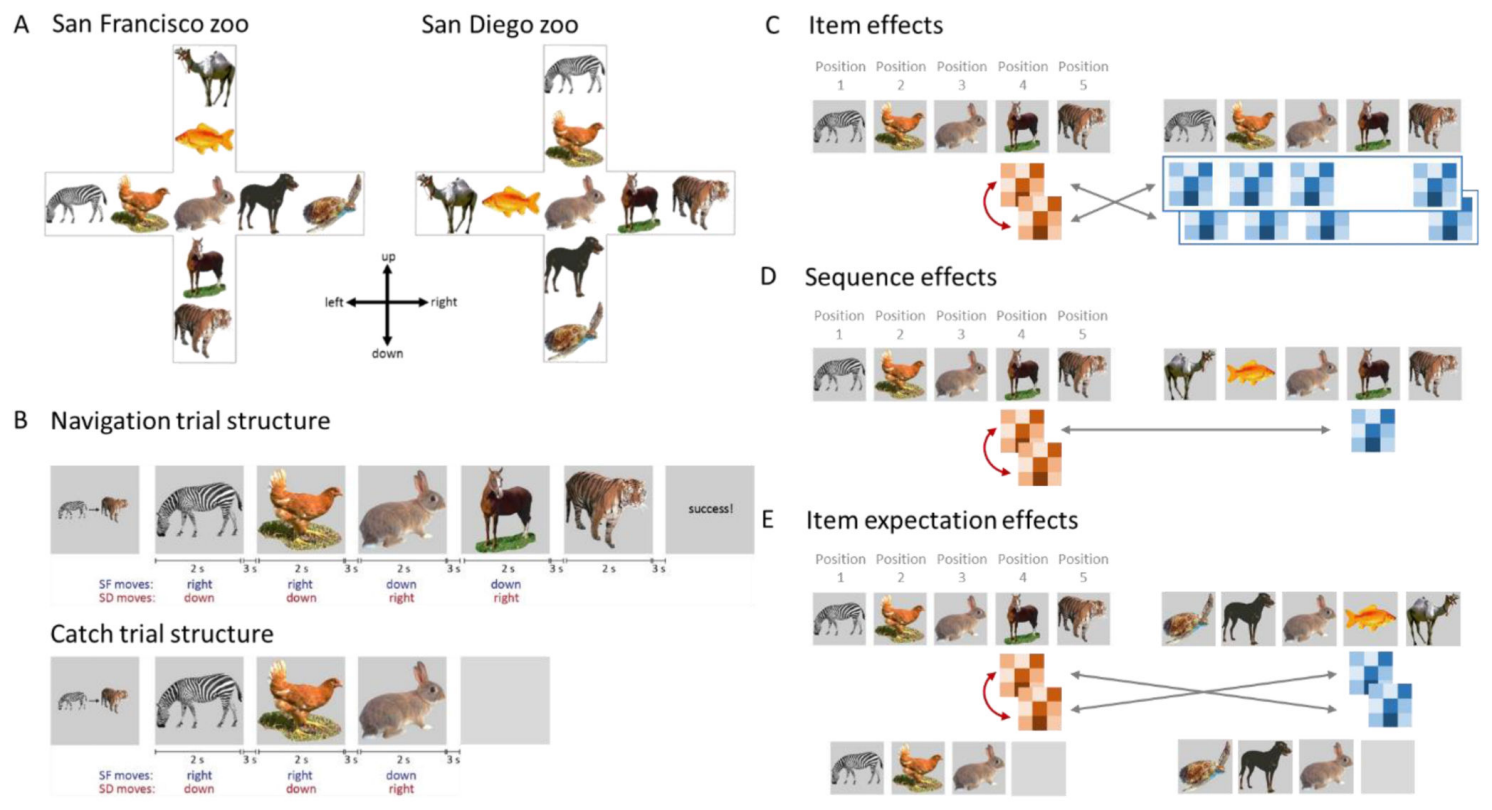
Experimental design and analysis. A) Participants learned the spatial layout of 9 animals in two distinct zoos. B) During fMRI, participants navigated between two animals, seeing a predictable sequence of images. In a quarter of trials, the sequence ended early where a specific item was expected, but instead a blank screen was shown. C) Item effects were established by comparing activity patterns when seeing the same item in the same sequence and same zoo (red arrow), with patterns for different items within the same sequence context and zoo (grey arrows). D) Sequence effects were established by comparing activity patterns between the same items seen in the same sequence, against patterns when the same item was seen in a difference sequence, while controlling for position (position 4 only used) and zoo. E) Item expectation effects were established by comparing activity patterns between the blank period (catch trial) and the item that was expected given the sequence context (red arrow), against activity patterns for different blank and seen items (grey arrows).

To explore these issues, we conducted an fMRI study where participants navigated through a learned space and saw predictable sequences of objects (Figure 1AB). Critically, a quarter of the sequence trials terminated early, where a specific item was expected, but instead a blank screen was presented. Using pattern similarity analysis, we ask (1) which regions represent item information, (2) are item representations specific to the sequence context, and (3) do these regions also represent information about expected items, even if not shown. To test how the hippocampus interacts with other regions, we employed multivariate representational connectivity (Kriegeskorte et al., 2008; Anzellotti and Coutanche, 2018; Pillet et al., 2020) to test whether the representational structure in the hippocampus at one point in the sequence related to a later point in the sequence, providing evidence that hippocampal representations support the reactivation of expected future activity patterns in cortex.

## Materials and Methods

### Participants

Thirty healthy individuals participated in the study. All participants had normal or corrected-to-normal vision and were right handed. Data from one participant was excluded due to technical complications with the fMRI scanner, one subject was excluded due to incomplete behavioral data, two subjects were excluded due to poor behavioral performance in the scanner (defined as falling below trained criterion, 85% correct, in the scanner), and one subject was removed from the scanner before the experiment was completed. Prior to data analysis, to ensure data quality, we conducted a univariate analysis to look at motor and visual activation during the task compared to an implicit baseline. Two subjects showed little to no activation in these regions and were excluded from further analysis. This resulted in twenty-three participants reported here (11 male, 12 female, all right handed). Written informed consent was obtained from each participant prior to the experiment, and the study was approved by The Institutional Review Board at the University of California, Davis.

### Experimental Design

Stimuli: The stimuli consisted of nine common animal images. Each animal was represented by a color photograph, presented in isolation on a grey background. The nine animals were positioned into a cross maze with two animals per arm and one in the central location, creating the first zoo called ‘San Francisco Zoo’. A second zoo was created, ‘San Diego Zoo’, by mirror-reversing and rotating 90 degrees anti-clockwise one zoo map to create the other. Therefore, both zoos contained the same animals with the same transitional structure but a different global layout (Figure 1A).

Training: Participants initially underwent a training session in order to learn the animals and their locations within the two zoos. To achieve this, participants completed map construction, exploration and navigation trials for one zoo, with the process then repeated for the second (zoo order counter-balanced across participants). During map construction, participants were shown the zoo layout and asked to arrange the animal images shown on screen into the correct positions. During exploration, participants were shown the animal at the center of the maze and were free to move up/down/left/right to see how moving in different directions resulting in seeing a different animal. After making 9 moves, the exploration reset to the center animal to begin again. Exploration continued until each animal was seen at least four times. Next, participants navigated between positions in the maze. They were shown a cue image indicating a start and a goal animal, followed by seeing the start animal. The participant had to select the correct moves to reach the goal animal. Start and goal animals were always located at the end points of an arm, and participants had a maximum of four moves. If participants did not reach criterion during navigation, they repeated map construction and navigation. After completing training with one zoo, the procedure was repeated with the second zoo. After successfully completing training with both zoos, participants completed a final navigation task including trials from both zoos and presented with the same structure and timings of the fMRI navigation task (see below). All training and navigation tasks were presented using Psychtoolbox and Matlab.

fMRI: During fMRI scanning, participants performed the navigation task for both zoos in a blocked fashion across six scanning runs. In each run, participants were told which zoo they were in and completed eight navigation trials for one zoo before switching to the other zoo. A navigation trial consisted of seeing a cue screen showing the start and goal animal for 3 seconds, followed by a blank screen for 3 seconds. The start animal was then displayed for 2 seconds followed by a 3 second blank screen. After a response, the relevant animal was shown for 2 seconds followed by a 3 second blank screen, with the process continuing for a maximum of four moves. After four responses were made, the participant was shown a feedback screen. The participant was required to make their response within 2 seconds of the animal appearing, otherwise the move was judged as incorrect and they were shown a text screen indicating ‘wrong move’ for 2 seconds followed by a 3 second blank screen, and the animal was shown again.

Participants competed navigation trials for the twelve possible start and goal animal combinations in each zoo, with each navigation trial being repeated 3 times resulting in 72 full navigation trials. A total of 24 catch navigation trials were included where each navigation trial was terminated early, after the third animal (the central animal if correct responses were made), and instead of seeing the fourth animal, participants saw an additional blank screen lasting six seconds before a new trial began. The order of zoos was counterbalanced both across runs and between participants.

### Scanning acquisition

MRI data were acquired on a 3T Siemens Skyra MRI using a 32-channel head coil. Anatomical images were collected using a T1-weighted magnetization prepared rapid acquisition gradient echo (MPRAGE) pulse sequence image (TR = 1800ms; TE = 29.6 ms; flip-angle = 7 degrees; 1 mm^3^ isotropic voxels; 208 axial slices, TR=2100ms, TE=2.98ms, FOV = 256mm). Functional images were collected with a multi-band gradient echo planar imaging sequence (TR = 1222 ms; TE = 24 ms; flip angle = 67 degrees; matrix=64x64, FOV=192mm; multi-band factor = 2; 3 mm^3^ isotropic spatial resolution).

### Data Preprocessing

Preprocessing used SPM12 (https://www.fil.ion.ucl.ac.uk/spm/). Functional images underwent slice time correction, spatial realignment and smoothing using a 4mm FWHM Gaussian kernal. To detect fast motion events, the ART repair toolbox (Mazaika et al., 2009) was used. These spike events were used as nuisance variables within the GLMs. Single item beta images were obtained by running a separate GLM for each object (LSS model; Mumford et al., 2012). For each GLM, the item of interest was entered as a single regressor with 1 event, with an additional regressor for all other events. All events were modelled as a 2 second boxcar and convolved with a canonical HRF. Additional regressors were included for each spike event identified, 12 motion regressors (6 for realignment and 6 for the derivatives of each of the realignment parameters), and a drift term using a 128s cutoff. This resulted in five beta images per full navigation trial (e.g. zebra, chicken, rabbit, horse, tiger) and four beta images for each catch navigation trial (e.g. zebra, chicken, rabbit, omitted item).

### Pattern Similarity Analysis

ROI PS: Our initial analysis focused on anatomical regions of interest (ROIs) in early visual cortex and the hippocampus, both of which are suggested to support sensory expectations and predictions (e.g. Hindy et al., 2016; Kok and Turk-Browne, 2018). A V1/V2 region was created from the functional atlas of visual cortex developed by Rosenke et al. (2021), where V1 and V2 were combined into a single region, and inverse normalized to native space. Probabalistic maps of the hippocampal head, body and tail were obtained from the multistudy group template (Yushkevich et al., 2015). These maps were warped to MNI space using DARTEL and thresholded at 0.5. The resulting maps were then reverse normalized to each participants native space using Advanced Normalization Tools. The anterior hippocampus was defined as the hippocampal head, and the posterior hippocampus as the combined body and tail sections. This division closely follows recommended anterior–posterior divisions (Poppenk et al., 2013). An additional set of ROIs were tested, with the perirhinal cortex (PRC) and parahippocampal cortex (PHC) defined from the multistudy group template (Yushkevich et al., 2015), and the temporal pole and PMC (combined precuneus and posterior cingulate cortex) were generated using FreeSurfer (version 6) and warped to each participants native space.

Pattern similarity was used to test three issues, if regions represented (1) the currently viewed item, (2) the item in a specific sequence context, and (3) the item that was expected but never shown. Our analyses focused on the item in position 4 of the sequence context, first because position 4 items are always preceded by the same image in all sequences (i.e. a rabbit), meaning any impact of the preceding item on voxel patterns due to autocorrelation is controlled for. Second, one quarter of position 4 trials were omitted with the trial ending early, allowing us to study the impact of expectations through these catch trials. Third, position 4 is also situated after a key decision point (position 3), where it is possible to see multiple different animals following the central item (the rabbit), with the decision made at this point determines the next image. Therefore, position 4 allows us to both examine item-level effects, while controlling for autocorrelation and recent visual effects, and predictive effects generated from the central decision point.

For item effects, we contrasted pattern similarity (PS) based on repetitions of the same position 4 item against when the items were different (Figure 1C). In order to control for sequence effects, PS was restricted to trials from the same sequence context and zoo, meaning that we are asking whether the items are dissociable within a specific sequence (although this does not control for the position within the sequence). Same-item PS was calculated between all possible pairs of the same item-sequence-zoo items and averaged. Different-item PS was calculated between pairs of items that were not the same, but were from the same sequence context and zoo, before being averaged.

Item expectation effects were tested in the ROIs showing significant item effects, and were based on PS between the expected-but-omitted beta image and the beta image of trials where the same item was seen. All omitted items were at positions 4 (Figure 1E). Specifically, PS was calculated between each omitted item and the position 4 trials where the same item was seen. To do this, PS was first calculated between an item and an omitted item where they are matched for item, sequence context and zoo, before calculating PS values when items are matched for item, but not sequence context and/or zoo. This results in four PS values which are averaged to give an overall PS between an item and when an item was expected-but-omitted. Importantly, this value includes PS values containing all visual histories that converge on that position 4 item. Baseline PS values were calculated when the item and omitted item were due to be different items, which will also have different visual histories. This ensures that expectation effects are matched for the past items in the sequence, as all item expectation PS values will include data with all sequence histories.

In order to test for effects of sequence context (Figure 1D), first, PS was calculated using the position 4 items between pairs of trials that shared the same item, sequence context and zoo. Then we calculated PS when pairs of items shared the same item and same sequence, but from the other zoo. These PS values were then averaged across the two zoos giving a PS reflecting the same items in the same sequence. These PS values were contrasted with PS for the same-items when in a different sequence (averaged across zoo). We chose to average the PS values across the two zoos because here we are interested in sequence-level contexts, and not potential global zoo differences, meaning our analyses focused on differences between the sequences without considering the two zoos (additional control analyses show no significant or marginal effects of zoo on sequence effects).

All pattern similarity values were calculated between pairs of items using Pearson’s correlation, excluding items where an incorrect response was made, and excluding pairs of trials that occurred in the same scanning run (Mumford et al., 2012). Pattern similarity was calculated using all grey matter voxels within each ROI.

Multivariate connectivity: We used a measure based on representational connectivity analysis (Kriegeskorte et al., 2008; Anzellotti and Coutanche, 2018; Pillet et al., 2020), an approach where PS in one region is correlated with PS in another region. However, here we adapted this approach to assess connectivity both between regions and from different positions in the sequence. This allowed us to test the degree to which PS in one region related to PS in another region at a different position in the trial. Here, we tested whether PS in the hippocampus at position 3 in the sequence was related to PS at position 4 in cortical regions that showed item expectation effects. To do this, we used a partial correlation analysis, whereby hippocampal PS from position 3 was correlated with position 4 PS from a second region, while controlling for PS from the second region at position 3. The analysis tells us if past information in the hippocampus can explain future information in cortex, over and above that explained by past information in that same cortical region. Our analysis focused on PS between trials from the same sequence context, and same zoo (excluding across sequence/zoo PS), and only included PS values calculated between trials in different scanning runs.

### Statistical Analysis

Pattern similarity values for item, sequence context and expectation effects were calculated for each participant and each ROI, and tested using paired-samples t-tests or one-sampled t-test against zero. An FDR correction was applied to p-values to control for the number of ROIs tested. Multivariate connectivity was calculated for each participant and tested using a one-sample t-test against zero. In addition to this frequentist approach, we analysed all contrasts using Bayesian one or two-sampled t-tests in JASP (version 0.14.1), where the null was defined as an RSA effect of 0, with a Cauchy prior width set to 0.707. Bayes factors are reported, indicating the ratio of evidence supporting our hypothesis compared to the null hypothesis.

## Results

### Behavioral learning and task performance

The experiment was conducted in two parts, a pre-fMRI learning session and a sequence navigation task during fMRI. During the pre-fMRI session, participants were required to learn the identities and locations of nine different animals in two related zoos (Figure 1A). During the fMRI scanning session, participants completed six runs of navigation trials, where each run consisted of blocks of eight trials from each zoo. In each zoo, there were 12 different navigation trials, with each full sequence of five animal items being repeated three times. As the same animals were found in both zoos, with the same transitions, identical visual sequences were seen in both zoos. During scanning, participants showed a high level of performance for both zoos (San Francisco: mean = 94.3%, SD = 6%, San Diego: mean = 95.0%, SD = 5%) with no statistical differences seen between them (t(22) = 1.11, p = 0.28). This high level of performance indicates that the sequences were well-known and therefore participants would be able to predict what animal was to appear next due to the sequence context (although they were not instructed to do this).

### Item, sequence and expectation effects in the early visual cortex and hippocampus

Our initial analysis of the fMRI data focused on the early visual cortex and the hippocampus, both of which are suggested to support sensory expectations and predictions (e.g. Hindy et al., 2016; Kok and Turk-Browne, 2018). Using multivariate pattern similarity analysis, we tested the extent to which these regions represented information about: (1) the currently viewed item, (2) the specific sequence context, and, (3) the next item that was expected in the sequence.

**Figure 2:**
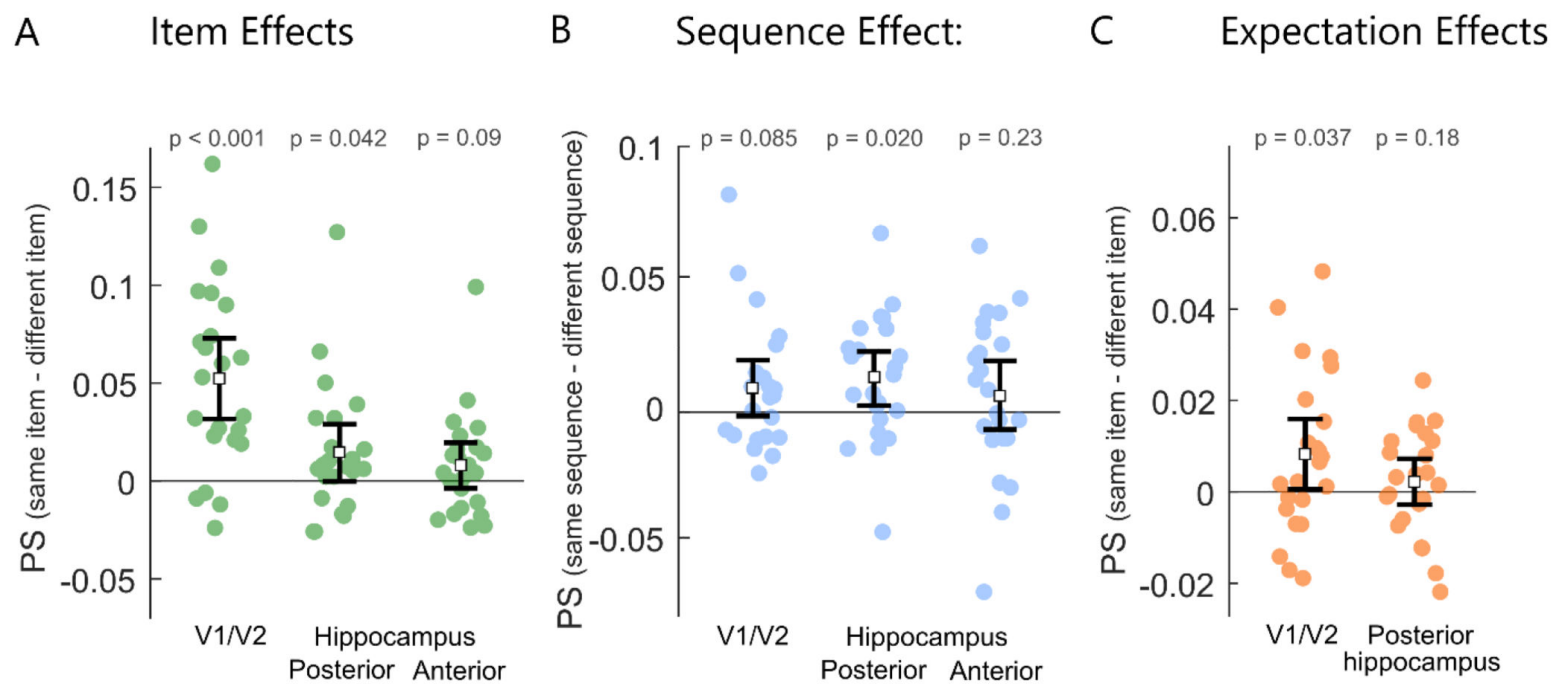
Pattern similarity results for early visual cortex and the hippocampus. A. Item effects showing difference in pattern similarity between same item pairs and different item pairs. FDR correction applied to p-values. B. Sequence effects showing changes in pattern similarity for same item pairs in the same sequences compared to seeing the same item in different sequences. C. Item expectation effects showing the difference in pattern similarity when the same item was omitted or seen, and when the omitted and seen item were different. FDR correction applied to p-values. Error bars show 95% confidence intervals around the mean.

We first determined if early visual cortex and the hippocampus were sensitive to information about the currently viewed item. To do this, we contrasted voxel pattern similarity (PS) for repetitions of the same item and compared this to PS between different items (Figure 1C). In order to control for sequence effects, PS was restricted to trials from the same sequence context and zoo, meaning that we are asking whether the items are dissociable within a specific sequence context. Significant item effects were seen in V1/V2 (mean = 0.052, t(22) = 5.26, p < 0.0001; BF_10_=1720 indicating very strong evidence for an item effect) and the posterior hippocampus (mean = 0.011, t(22) = 2.02, p = 0.042; BF_10_=2.3, anecdotal evidence for an item effect), but not the anterior hippocampus (mean = 0.010, t(22) = 1.37, p = 0.09; BF_10_=0.9, anecdotal evidence for the null hypothesis; Figure 2A).

These item-level representations may reflect the visual appearance of the object, however, given past research showing the hippocampus is sensitive to contextual sequence information (Ezzyat and Davachi, 2014; Hsieh et al., 2014), we next asked if these item effects were dissociable across the different sequence contexts. We compared PS between the same-items in the same sequence, to PS for the same-items when found in different sequences. As our analysis only included items in position 4, and PS is calculated between same items pairs, any differences we see are driven purely by information pertaining to the sequence context, and not by visual details of item or temporal order. Significant sequence effects were found in the posterior hippocampus, where patterns were more similar for same-items from the same sequence (mean = 0.041) compared to same-items across different sequences (mean = 0.030; t(22) = 2.52, p = 0.0196; BF_10_=3.1, moderate evidence for sequence effects). No significant sequence effects were observed in V1/V2 (t(22) = 1.81, p = 0.085; BF_10_=0.96, anecdotal evidence for the null hypothesis) or the anterior hippocampus (t(22) = 1.22, p = 0.23; BF_10_=0.42, anecdotal evidence for the null hypothesis), although our analyses also do not support the absence of a sequence effect in these regions. These results show that the posterior hippocampus not only represents information about the current item, but that these representations are further reflective of the specific sequence context the item occurred in.

In the above analysis, we characterized representations of presented objects within a learned sequence. In well-learned sequences, upcoming items are known, and according to predictive models of perception, being able to predict upcoming items should impact neural processing by generating expectations about what is about to happen (Bar, 2004; Trapp and Bar, 2015; de Lange et al., 2018; Turk-Browne, 2019). To test this hypothesis, we focused our next analyses on catch trials (see Figure 1D), in which each sequence was terminated early, such that, after position 3, a blank screen was shown for six seconds, followed by the onset of the next navigation trial. In other words, on every catch trial, there was no motor response or external visual stimulation, and the preceding image was matched for all sequences. Thus, any representational content during a catch trial would be expected to be driven by memory-driven predictions in the absence of bottom-up input.

To test whether the regions that were sensitive to perceived items also carried information about expected items in the absence of sensory input, we assessed PS between presented items and activity when an item was expected but omitted from the sequence. PS was calculated between items and the catch trials when they were the same item, and compared to when the presented and catch trials were different (Figure 1D). Significant item expectation effects were seen in V1/V2 (mean = 0.008, t(22) = 2.25, p = 0.037; BF_10_=3.3, moderate evidence for expectation effects) but not the posterior hippocampus (mean = 0.002, t(22) = 0.58, p = 0.19; BF_10_=0.51, anecdotal evidence for the null; Figure 2C). Our analyses clearly show that activity patterns in early visual regions are not only shaped by the bottom-up visual input, but that contextually-predicted item information is reactivated which matches the expected visual input.

Together, our analysis of early visual cortex and the hippocampus reveals that while item information is present in both regions, item representations in the posterior hippocampus were further modulated by the sequence the item was in, and in early visual cortex there was evidence of item patterns being reactivated when they were expected, but failed to appear.

### Item and expectation effects in the posterior medial cortex

As discussed earlier, a wider network of regions beyond the hippocampus have been implicated in memory-guided predictions and contextual reactivations (Bar and Aminoff, 2003; Lee and Kuhl, 2016; Livne and Bar, 2016; Jonker et al., 2018; Caplette et al., 2020; Long and Kuhl, 2021). The PM network is functionally connected to the posterior hippocampus, and associated with reactivation of contextually-relevant object information, whilst the AT network is connected to the anterior hippocampus and is thought to represent item information (Ranganath and Ritchey, 2012). As such, we next repeated our analysis of item, sequence and expectation effects across regions in the PM network - parahippocampal cortex (PHC), posterior medial cortex (PMC; precuneus/posterior cingulate cortex) and the angular gyrus - and the AT network - the temporal pole and perirhinal cortex (PRC).

**Figure 3:**
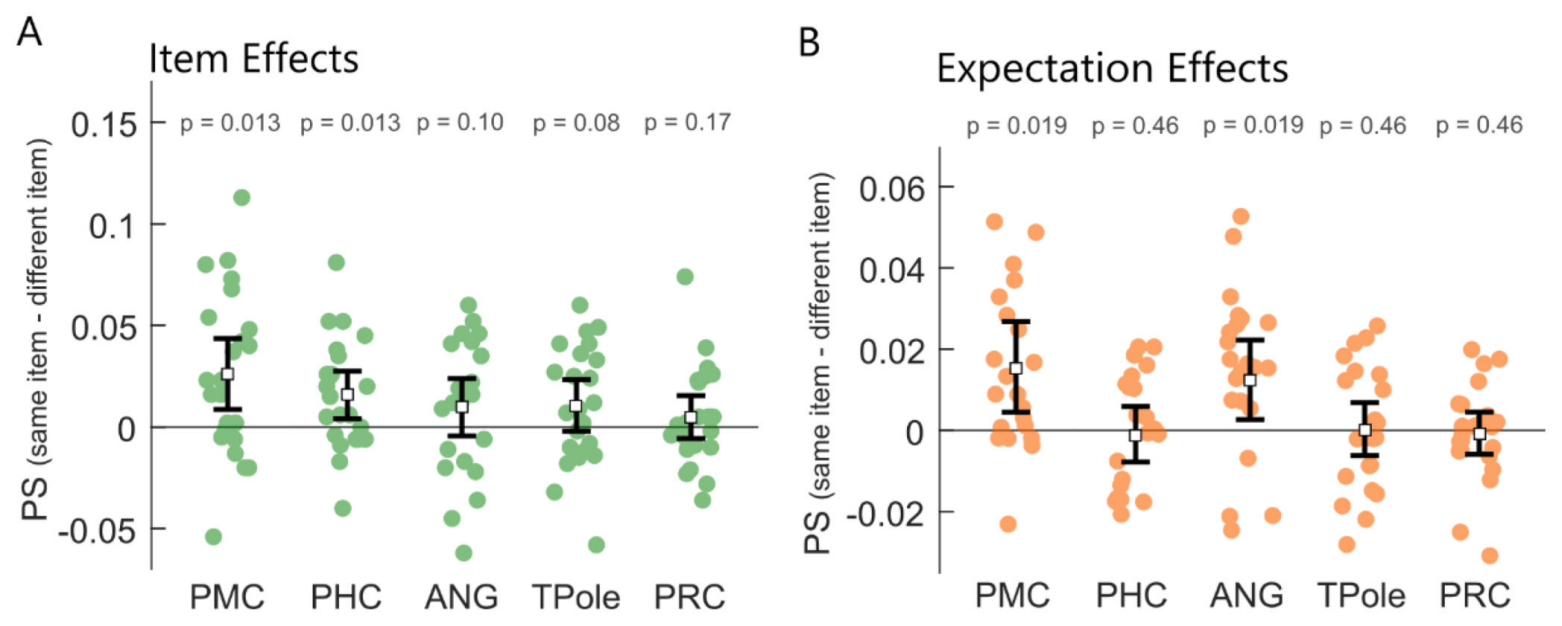
Pattern similarity results for regions of the PM and AT networks. A. Item effects showing difference in pattern similarity between same item pairs and different item pairs. FDR correction applied to p-values. B. Item expectation effects showing the difference in pattern similarity when the same item was omitted or seen, and when the omitted and seen item were different. Error bars show 95% confidence intervals around the mean.

Item effects were calculated by comparing PS between same-item pairs with PS for different item pairs, within the same sequence context. Significant item effects were seen in PMC (mean = 0.026, t(22) = 3.11, p = 0.0128; BF_10_ = 17.3, strong evidence for an item effect) and the PHC (mean = 0.016, t(22) = 2.80, p = 0.013; BF_10_ = 9.5, moderate/strong evidence for an item effect), but not the angular gyrus (mean = 0.010, t(22) = 1.44, p = 0.10; BF_10_ = 0.98, anecdotal evidence for a null effect), temporal pole (mean = 0.11, t(22) = 1.74, p = 0.080; BF_10_ = 1.5, anecdotal evidence for an item effect) or PRC (mean = 0.005, t(22) = .95, p = 0.17; BF_10_ = 0.5, anecdotal evidence for a null effect; Figure 3A). This suggests that in addition to the early visual cortex and posterior hippocampus, regions of the PM network – the PMC and PHC - also represent the currently viewed item during navigation.

We next asked whether these regions represented the same items in a distinct manner across sequence contexts by comparing PS for same-item pairs from the same sequence, against same-item pairs across different sequences. This analysis revealed no significant sequence effects (all p’s > 0.05; ANG, TPole and PRC show BF_10_ < 0.3, moderate evidence for the null). Finally, we tested whether the regions that showed item effects also showed effects of expected items in the absence of bottom-up visual input by comparing PS for when items and catch trials were the same item, to when the presented and catch trials were different. Significant item expectation effects were in the PMC (mean = 0.016, t(22) = 2.96, p = 0.0187; BF_10_ = 11.6, strong evidence for an expectation effect) but not the PHC (mean = -0.001, t(22) = 0.27, p = 0.46; BF_10_ = 0.18, moderate evidence for the null; Figure 3B). Additional exploratory analyses across the remaining regions showed an item expectation effect in the angular gyrus (mean = 0.012, t(22) = 2.70, p = 0.0187; BF_10_ = 7, moderate evidence for an expectation effect), but not temporal pole (mean = 0.001, t = 0.10, p = 0.459; BF_10_ = 0.24, moderate evidence for the null) or perirhinal cortex (mean = -0.001, t = -0.26, p = 0.459; BF_10_ = 0.18, moderate evidence for the null; Figure 3B).

Overall, our data point to a representation of the current item in a network of regions in early visual, PM and the posterior hippocampus, with item representations in the posterior hippocampus being further specific to the sequence context. Crucially, representations in early visual cortex and the PMC reflect the expected item in the absence of any bottom-up input, suggesting they are a site of top-down effects based on contextual expectations. While our results primarily point to effects in the primary visual cortex, posterior medial cortex and posterior hippocampus, we cannot rule out item and expectation effects in other regions, given our Bayesian analysis largely do not support the null hypothesis, meaning that we cannot make any conclusions about the presence or absence of item or expectation effects in other regions.

### Hippocampal and cortical interactions support reactivation

**Figure 4:**
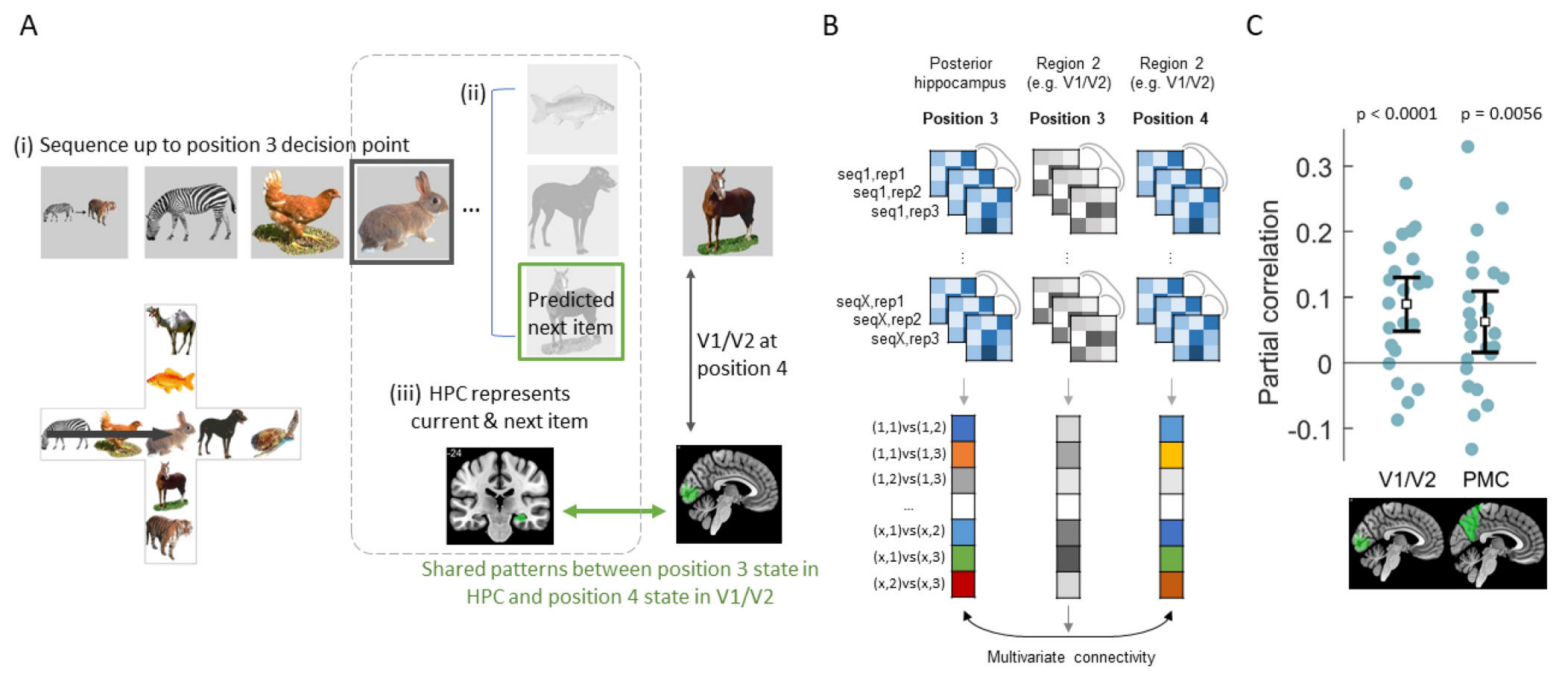
Cross region and position multivariate connectivity. A. (i) Participants navigate the sequence up to position 3, the central decision point of the cross-maze. (ii) From here it is possible to see 3 different animals, one of which is the correct next item for this sequence. (iii) If hippocampal representations are predictive of future states, then the hippocampal representation at position 3 will also contain some information about the correct future state (position 4). If so, representations in the hippocampus at position 3 should share information with the patterns in cortex at position 4, where item representations and expectation effects are seen. This is tested using multivariate connectivity. B) First, posterior hippocampal activity patterns are extracted for each rabbit item (position 3) for each repetition of each sequence. Pattern similarity is calculated between each repetition of the same-sequence pairs, resulting in a correlation vector, analogous to an unwrapped representational similarity matrix, but limited to same-sequence pairs. Following this, similarity is calculated between same sequence/item-pairs at position 4 for repetitions from the same sequence taken from a second region (e.g. V1/V2), producing a second similarity vector. Multivariate connectivity is calculated as the partial correlation between the two similarity vectors from the two regions/positions while controlling for the similarity of region 2 at position 3. C) Posterior hippocampus was significantly correlated with later similarity patterns in V1/V2 and PMC. Error bars show 95% CI around the mean.

An important question arising from our results, is by what mechanism are visual details of the items being reactivated in early visual cortex and PMC? Motivated by the role of the hippocampus in representing sequence knowledge, cortical reinstatement and the prediction of future states (Eichenbaum and Fortin, 2009; Hindy et al., 2016; Stachenfeld et al., 2017; Kok et al., 2020; Turk-Browne, 2019), we next tested the hypothesis that hippocampal pattern information related to future information states in visual cortex and PMC - regions showing item expectation effects, which may suggest the hippocampus supports the prediction and reactivation of sensory patterns in cortex. We reasoned that, if this is the case, the fidelity of a hippocampal sequence representations at one state (as indexed by PS across same-sequence pairs) should be predictive of the fidelity of the cortical sequence representation at the next position. We focused on position 3 in the sequence, as this is a critical decision point where one must choose amongst three possible states. Thus, we expected that hippocampal predictions about future states may be enhanced at this decision point (Johnson and Redish, 2007; Singer and Frank, 2009; Pfeiffer and Foster, 2013). If so, representations in the hippocampus at position 3 should share information with the patterns in cortex at position 4, where item representations and expectation effects are seen (Figure 4A).

To test this, we employed a multivariate connectivity approach based on representational connectivity analysis (Kriegeskorte et al., 2008; Anzellotti and Coutanche, 2018; Pillet et al., 2020). Representational connectivity analysis asks if PS in one region is correlated with PS in another region, with a significant correlation indicating cortical representations are shared between the regions (Pillet et al., 2020). Here, we adapt this approach to assess connectivity between regions and from different positions in the sequence. We first calculated hippocampal PS between same sequence pairs at position 3, which is the central decision point in the sequence (where all items are rabbits; Figure 4B). We next calculated PS in cortical ROIs, V1/V2 and PMC, in same sequence trial pairs at position 4. These ROIs were specifically chosen as they showed item expectation effects, suggesting they are a target for item reactivation. In order to ensure that any connectivity between the hippocampus at position 3 and cortex at position 4 was not due to overall shared information between position 3 and position 4, we further controlled for the influence of position 3 PS from the same cortical region using partial correlation. This analysis thus allows us to ask whether information in the hippocampus at position 3 is related to cortical information at the next point in the sequence, over and above what position 3 cortical responses could account for. We find significant partial correlations between position 3 representations in posterior hippocampus and position 4 representations in V1/V2 (mean p*_xy_* = 0.089, t(22) = 4.51, p < 0.0001; BF_10_ = 355, very strong evidence for an effect) and PMC (mean p*_xy_* = 0.063, t(22) = 2.77, p = 0.0056; BF_10_ = 8.9, moderate/strong evidence for an effect; Figure 4C). These results show that hippocampal responses reflect information about the upcoming states that later emerge in early visual and PMC, and is consistent with a predictive role of the hippocampus in supporting cortical reinstatement of expected future items. As an additional exploratory analysis, we further tested for relationships between PS at position 3 in the posterior hippocampus and the PMAT regions at position 4. This revealed no additional significant relationships for PHC (p = 0.0604), ANG (p = 0.0792), TPole (p = 0.0922) or PRC (p = 0.2468).

## Discussion

Here we asked how the hippocampus, PM network and early visual cortex interact to support the prediction and reactivation of sensory details in cortex. Our results revealed that the hippocampus, posterior medial cortex and early visual regions, work together to represent, predict and reactivate sensory details of future events. After learning the locations of animals within a cross maze structure, participants moved through the maze during fMRI, seeing predictable sequences of images. Importantly, in our paradigm, one quarter of the sequences ended early, meaning that the next image in the sequence was expected but not shown, allowing us to investigate the nature of top-down expectations in the absence of visual input. Pattern similarity (PS) analysis revealed complementary roles for the hippocampus and neocortical areas. Specifically, the posterior hippocampus carried information about items and sequence contexts, whereas visual cortical areas carried information about the currently processed item, as well as item expectations. Furthermore, we found that the fidelity of hippocampal PS predicted subsequent item-specific representations in early visual cortex and the PMC. These findings show that hippocampal representations are used to generate expectations of future inputs via top-down modulation to the neocortex.

Our analysis of item effects contrasted same item and different item trials while controlling for sequence context and zoo. However, by controlling these factors, the position of the item in the sequence is not controlled for. In relation to our findings of item effects in V1/V2 and PMC, it is unlikely that position drives these effects because we find item expectation effects in these same regions in an analysis which does control for position effects (item expectation effects can only be studied at position 4). In terms of the posterior hippocampus, while it is possible position information could contribute to item effects, we find this unlikely given that we do find sequence effects in this region (in a contrast that controls position) and previous similar paradigms have found the hippocampus represents item-in-sequence information and not solely position information (Hsieh, et al., 2014). Importantly, as our analysis of multivariate connectivity assess PS across positions, and item expectation effects control for position, our main findings are not limited by any potential impact of position on our item effects.

### Hippocampal memories guide the reactivation of upcoming sensory details

Using multivariate connectivity, we tested if the representational similarity structure of the hippocampus related to representational similarity in V1/V2 and PMC, with the inference being that correlated states between regions indicates representations are shared between the regions (Pillet et al., 2020). Importantly, we calculated multivariate connectivity between the hippocampus at position 3 in the sequence (a rabbit image in all sequences) and V1/V2 and PMC at position 4, meaning that in addition to testing for shared representations across regions, we further tested the idea that information is shared between past hippocampal responses and future cortical responses. We observed a significant relationship between the posterior hippocampus at position 3 with both V1/V2 and PMC at position 4. These results argue that the hippocampus is a top-down source of predictive effects. Note that this effect cannot be driven by a concurrent strong representation of the current item in both regions, as our analysis controls for effects in the cortical regions at position 3. This means there is some element of representational similarity in the hippocampus at position 3 that can explain future representational similarity seen at position 4 in cortex, over and above that explained by the cortical regions at position 3 themselves. These results align with the view that hippocampal memories guide prediction of upcoming sensory events.

How might these predictive effects come about? One line of research to illustrate the link between the hippocampus and prediction, is that of statistical learning. Studies of statistical learning argue that the hippocampus enables us to learn the structure of our environment, which can then be used to predict upcoming events and help guide behavior (Schapiro et al., 2014; Kourtzi and Welchman, 2019; Turk-Browne, 2019; Sherman et al., 2020). Human fMRI data indeed points to the hippocampus for representing the temporal order of learned object sequences (Hsieh et al., 2014) and for predicting future states during navigation in learned environments (Brown et al., 2016). Further, hippocampal place cells have been shown to reactivate prospective future locations along a navigational path, a phenomenon termed preplay (Johnson and Redish, 2007; Lisman and Redish, 2009). Together with our results, this points to a mechanism whereby the hippocampus is engaged in predicting upcoming events, through reinstating learned details.

This leads to a question of why we did not directly observe item expectation effects in the hippocampus yet found evidence that hippocampal patterns at position 3 related to future cortical patterns at position 4? Previous research might indicate that hippocampal patterns reflect the expected stimulus, despite it being omitted, during a tasks where a cue is explicitly predictive of a specific shape (Kok et al., 2020). Additionally, when learning such statistical regularities, the hippocampus has been shown to represent the upcoming predictions over the current input (Sherman et al., 2020). Our analyses focussed primarily on items during the navigation period of the trial, which was preceded by a cue indicating the sequence identity. Using the same data as reported here, Crivelli-Decker et al., (2021) did in fact show that hippocampal patterns during the cue indicated the identity of the following (or expected) sequence. This shows that the hippocampus is representing predictions of future states in the current data, in agreement with our representational connectivity analysis. However, the lack of expectation effects in the hippocampus could suggest that the dynamics of hippocampal and cortical interactions might shift between a cue-item prediction paradigm and our more complex navigation paradigm involving multiple items in succession.

Learning paradigms have further been employed to reveal the instantiation of visual predictions, where after learning sequences of visual gratings, the orientation of an expected grating can be decoded from early visual cortex (Luft et al., 2015). In conjunction with our data, these studies point to the hippocampus being a source of top-down modulation on early visual regions. Predictions about upcoming items could be reactivated in the hippocampus, through pattern completion (McClelland et al., 1995), with information about the expected sensory details then reactivated in cortex. In humans, evidence is emerging linking hippocampal pattern completion to visual predictions (Hindy et al., 2016; Kok and Turk-Browne, 2018). For example, Hindy et al., (2016) used fMRI after participants learned cue-response-outcome associations. Using multivariate classifiers trained on either the full association or the outcome alone, and applied to cue-response trials, they showed that hippocampal subfields CA1 and CA2/3DG contained information about the full sequence of associations, while V1 and V2 contained information about the expected perceptual outcome. Further, Hindy et al., (2016) showed that hippocampal sequence decoding was related to visual cortex outcome decoding. These results parallel our hippocampal sequence effects and early visual cortex item expectation effects. However, much of the previous evidence focused only on the hippocampus and visual cortex, while prediction was also part of the task. Here, participants navigated through learned environments and saw sequences of objects during a task that does not emphasize prediction. This allowed us to establish the top-down nature of hippocampal representations with visual cortex, extending past research by showing top-down effects in contextually sensitive posterior medial cortex, and during a task that involved goal-directed navigation, rather than cue-outcome predictions. This last point suggests critical evidence that such predictive processes are engaged during more natural behaviors. Our data further adds to a broad literature highlighting a top-down modulatory role of the human hippocampus on visual cortex, and beyond, during memory-guided behaviors such as retrieval (Bosch et al., 2014; Staresina and Wimber, 2019; Barron et al., 2020), navigation (Sherrill et al., 2013; Watrous and Ekstrom, 2014; Bridge et al., 2017) and attention (Stokes et al., 2012; Aly and Turk-Browne, 2016a, 2016b; Günseli and Aly, 2020).

### Predicted sensory details are reactivated in early visual and PM cortex

A critical question we addressed was whether regions represented expected items in the absence of any bottom-up input, finding that both V1/V2 and the PMC showed item expectation effects. This was possible due to our catch trials, where after position 3 in the sequence, the final two items were omitted and replaced by a blank screen. This meant that we could evaluate representations of what was expected, but did not appear. Such expectation effects require the retrieval of sequence information based on learned experiences, and the reactivation of the expected sensory patterns.

Previous studies have reported the reactivation of expected sensory details in primary visual regions for abstract stimuli (Alink et al., 2010; Eagleman and Dragoi, 2012; Kok et al., 2012, 2014), with our results showing this generalizes to complex meaningful items. Going beyond these studies, we revealed item expectation effects outside of primary visual cortex - in the PMC. The design of our study did not permit us to dissociate between visual and PMC representations, though the extant literature speaks to this issue. For example, recent fMRI research shows the PMC becomes more engaged when item predictions can be made from a preceding visual scene. Caplette et al., (2020) showed visual scenes followed by objects which were either predictable given general knowledge about a scene (e.g. a beachball is consistent with a beach scene) or the object was not predictable. They showed increased precuneus activity for predictable objects, suggesting the precuneus is integrating information about the contextual predictions and the object. In our paradigm, like the early visual cortex, the PMC showed expectation effects when an item was expected, but never shown. Such expectation effects in the PMC have also been observed during speech (Scharinger et al., 2016). Such findings suggest that the PMC might play a role in prediction that transcends sensory modalities, consistent with the idea that PMC representations may be relatively abstract or semantic in nature (Ranganath and Ritchey, 2012).

We also observed an item expectation effect in the angular gyrus. Past research indicates lateral parietal regions, such as angular gyrus, may preferentially represent retrieved content from memory in contrast to perceived stimulus details (Lee & Kuhl, 2016; Xiao et al., 2017; Favila et al., 2018). Our data suggests this may extend to the expectations and memory-guided predictions elicited by our study, where angular gyrus expectation effects were present, and not item effects. One limitation of our approach, is that we examine expectation effects through the relationship between seen and catch trials, requiring some shared information between them. However, it is possible that expectation related reactivations could be reflected by a transformation of the original perceptual experience, as might be the case for episodic memory reactivation (Xiao et al., 2017). How, and if, memory-guided reactivations reflect transformed states of the initial perceptual experience remains a key issue to be explored (Favila et al., 2020).

Item expectation effects are driven by the differentiation of activity patterns to different expected animals. Several lines of evidence show that when an item is highly predictable, there is an increase in stimulus decoding and decrease in activity magnitude (Alink et al., 2010; Kok et al., 2012). The stimulus-specific nature of our expectation effects, observed without the occluding impact of a stimulus, are consistent with models where predictions result in a sharpening of neural representations, resulting in reduced BOLD signals following top-down constraints (de Lange et al., 2018). In our study, the reactivated patterns were specific enough to distinguish between different expected animal images, yet what is less clear, is the level of detail and nature of information that was reactivated. As we hypothesize above, it is likely that low-level visual details are reactivated in early visual areas, and higher-level visual, semantic and contextual signals in the PMC.

Recent research has shown that hippocampal predictions when specific stimuli are expected depend on the complexity of the stimulus. Kok and colleagues (2020) have shown that while hippocampal responses contained information about the expected stimuli when they were abstract perceptual shapes, it did not do so when the expected items were orientation gratings. They suggest that, like with other regions within the MTL, stimulus complexity interacts with function. In our study, we also find evidence for a predictive function of the hippocampus with sequences of complex meaningful images, shown through our connectivity analysis, although find no evidence for item expectation effects in the hippocampus. However, other recent evidence using the same dataset shows the hippocampus does reflect future goals when the sequence is cued (Crivelli-Decker et al., 2021). Therefore, it will be important to determine how hippocampal predictions operate as a function of task state and stimulus complexity. Importantly, although expectation effects are often also seen in early sensory regions, participants in our paradigm know the exact stimulus that will appear. In real life situations, there can be much more uncertainty about what will be seen. In these cases, predictions might not be related to a specific perceptual stimulus, and instead they might be more conceptual in nature, with expectation effects limited to higher-level regions, such as the posterior ventral temporal cortex. These more general contextual predictions are indeed claimed to constrain responses in posterior ventral temporal cortex, where predictions (or perceptual hypotheses) generated in the orbitofrontal cortex constrain activity in posterior regions (Trapp & Bar, 2015). This raises the important questions of how real-world contextual predictions, and predictions of varying specificity modulates the strength and mechanism of hippocampal guided predictions.

It is long established that our past experiences impact our current perceptions. The current study provides novel insights into the mechanisms of how top-down expectations can influence the visual processing of objects. The current results advance our understanding of how the hippocampus and posterior cortical regions work together to support perceptual expectations and predictions of future states based on learned sequence contexts. Further, our results help to bridge between research on the cortical manifestation of expectations, and research on predictive and contextual representations in the hippocampus.

## Significance statement

Our visual world is a series of connected events, where we can predict what we might see next based on our recent past. Understanding the neural circuitry and mechanisms of the perceptual and memory systems that support these expectations is fundamental to revealing how we perceive and act in our world. Using brain imaging, we studied what happens when we expect to see specific visual items, and how such expectations relate to top-down memory signals. We find both visual and memory systems reflect item predictions, and moreover, we show that hippocampal activity supports predictions of future expected items. This demonstrates that the hippocampus acts to predict upcoming items, and reinstates such predictions in cortex.
